# Behaviors of Water Intake, Hydration Status, and Related Hydration Biomarkers among Physically Active Male Young Adults in Beijing, China: A Cross-Sectional Study

**DOI:** 10.1155/2022/9436186

**Published:** 2022-10-17

**Authors:** Na Zhang, Jianfen Zhang, Xing Wang, Yibin Li, Yi Yan, Guansheng Ma

**Affiliations:** ^1^Department of Nutrition and Food Hygiene, School of Public Health, Peking University, 38 Xue Yuan Road, Hai Dian District, Beijing 100191, China; ^2^Laboratory of Toxicological Research and Risk Assessment for Food Safety, Peking University, 38 Xue Yuan Road, Hai Dian District, Beijing 100191, China; ^3^College of Sports and Human Sciences, Beijing Sport University, 48 XinXi Road, Hai Dian District, Beijing 100084, China

## Abstract

Studies on the water intake of athletes in daily life are insufficient. The objective was to determine the water intake and hydration status among physically active male young adults. In this cross-sectional studies study, 111 physically active male young adults were recruited. The amount of daily total drinking fluid intake (TDF) among participants was recorded and evaluated in real time over 7 days using the “7-day 24-hour fluid intake questionnaire” (liq. In 7). The daily water intake from food (WFF) was calculated using the weighing, duplicate portion, and direct-drying method over 3 days. All urine samples over 3 days were collected, and urine biomarkers were determined. According to 24 h urine osmolality, the participants were divided into three groups with euhydration status, middle hydration, and hypo hydration statuses. Finally, 109 participants completed the study. The median daily total water intake (TWI), TDF, and WFF were 2701, ik1789, and 955 mL, respectively. Among participants, 17 participants (16%) were in euhydration status, 47 participants (43%) were in hypohydration, and 45 participants (41%) were in middle hydration. There were statistical significances in the 24 h urine volume, osmolality, urine specific gravity, and concentrations of K, Na, and Cl in different hydration statuses (*χ*^*2*^ = 28.212, *P* < 0.01; *χ*^*2*^ = 91.341, *P* < 0.01; *χ*^*2*^ = 47.721, *P* < 0.01; *χ*^*2*^ = 41.548, *P* < 0.01; *χ*^*2*^ = 46.863, *P* < 0.01; and *χ*^*2*^ = 40.839, *P* < 0.01). Moderate-intensity correlations were found between the TDF and 24 h urine volume, 24 h urine osmolality, 24 h urine Na concentration, morning urine osmolality, and morning urine Na concentration (*r* = 0.408, *P* < 0.01; *r* = -0.378, *P* < 0.01; *r* = −0.325, *P* < 0.01; *r* = −0.344, *P* < 0.01; and *r* = −0.329, *P* < 0.01). There were also moderate-intensity correlations between the TDF and 24 h urine osmolality, morning urine osmolality, and morning urine Na concentration (*r* = −0.365, *P* < 0.01; *r* = −0.371, *P* < 0.01; and *r* = −0.322, *P* = 0.01). Increased and higher moderate-intensity correlations were found between plain water and 24 h urine volume, 24 h urine osmolality, 24 h urine K and Na concentration, morning urine osmolality, and morning urine Na concentration (*r* = 0.374, *P* < 0.01; *r* = −0.520, *P* < 0.01; *r* = −0.312,*P* < 0.01; *r* = −0.355, *P* < 0.01; *r* = −0.446, *P* < 0.01; and *r* = −0.378, *P* < 0.01). Insufficient water intake and hypohydration were common among physically active male young adults. The amount and type of water intake were correlated with hydration status and urine biomarkers. The results could provide scientific and accurate references for the development of recommendations on water intake for athletes.

## 1. Introduction

Water is an indispensable nutrient for human survival and development. It is the main component of body tissue and also plays an important role in the process of various physiological functions [1–3]. Adequate and appropriate water intake is vital to maintain health [2]. Hypohydration caused by insufficient water intake may reduce cognitive function and increase the incidence of urinary system infections [4–10]. Although hyponatremia caused by excessive drinking water or heatstroke rarely occurs, it can cause headache, nausea, vomiting, and memory loss due to brain cell swelling, brain tissue edema, and increased intracranial pressure [1]. In severe cases, it can cause trance, coma, convulsions, and even death [1, 11]. Water demands are affected by many factors, including environment, age, dietary structure, physical activity. [1]. For athletes who have the habit of regular physical activity, their metabolism increases remarkably so as to support the contraction of skeletal muscle during physical activity. Heat is generated in this process and, to prevent damage caused by overheating, heat dissipation through sweating increases [1]. Therefore, athletes need a higher water intake due to increased water loss.

Ensuring adequate water intake among athletes is important for maintaining euhydration status. Several previous studies have shown that hypo hydration caused by insufficient water intake reduced physical activity abilities [12–17]. In addition, insufficient water intake and being in a hypohydration status during physical activity are harmful. When in hypo hydration during physical activity, the body's blood volume decreases, and the plasma osmotic pressure increases, resulting in an increase in the concentration of the extracellular fluid [18]. The water in the intracellular fluid of the whole body's cells is transferred outside the cells due to an increase in osmotic pressure. All these changes can lead a to shortage of systemic cell water [19–21]. Hypohydration of the body also leads to limitations in the normal heat dissipation function and an increase in core temperature [22] and even leads to heatstroke in severe cases [23]. In addition, hypohydration can also increase the risk of muscle cramping during physical activity [24]. Therefore, maintaining adequate water intake and euhydration status are of great significance to maintain the ability to physical activity and health.

However, the importance of water intake has not been paid enough attention to. There is still a lack of research on the water intake behavior of male athletes in their daily life. Some existing studies show that insufficient water intake and hypohydration may be common among athletes [25–27]. The results of one study conducted among male and female teenagers aged 11–14 years in Sports Federations in Brazil showed that the median daily total water intake (TWI) was 2.8 L/d, and 22% of participants did not reach the recommended amount for adequate TWI (2.1 L–2.4 L for children aged <14 years and 2.3 L–3.3 L for children aged ≥14 years) [28]. In another study, conducted among eight adolescent runners, the tap water intake was 1032 mL, while the flavored water intake was 1086 mL, and no significant dehydration was observed among them [29]. In a cross-sectional studies study, it was found that the soccer players recruited were slightly hypohydration evaluated using urine-specific gravity and body weight loss before training [30]. The development of recommendations on adequate water intake for athletes is important to promote sufficient water intake among athletes. Due to the lack of data on athletes' water intake, it remains unclear as to the extent that athletes are meeting current recommendations, as imprecise as these recommendations may be. Water demand is mainly affected by metabolism, gender, age, physical activity, temperature, dietary intake, and other factors, so water demand varies greatly. Water requirement not only varies greatly among individual, but also varies in different environments or physiological conditions of the same individual. Among many influencing factors, physical activity is closely related to water demand. When physical activity increases, the amount of water demand should increase. However, there is no scientific recommendation on water intake for populations at different levels of physical activity. Studying and analyzing the behaviors of water intake and hydration state of physically active populations will help to formulate relevant water intake recommendations based on the level of physical activity. Thus, it is necessary to carry out relevant studies.

Hydration status can be comprehensively evaluated using multiple indexes, including total water intake, body weight loss, serum osmolality, urine indicators (urination volume, urine osmolality, urine specific gravity, urine color, and a number of voids), saliva osmolality, tear osmolality, total body water, and some clinical symptoms and signs [31–33]. Serum osmolality can be used to evaluate acute and critical hypo hydration status, but was not sensitive to mild hypo hydration status [34]. Some studies showed that urine indicators vary according to water intake and can reflect the changes in hydration status in free-living adults with ad libitum access to fluids [34–37]. The results of most studies suggested that hydration status was best evaluated with the 24 h urine osmolality, and an evaluation criterion for hydration status was proposed [3, 38, 39]. Some studies suggested that there were associations between water intake, hydration-related urine, and blood biomarkers [3, 40]. More studies should be conducted to explore the strength of the relationship between water intake and hydration biomarkers, to develop a highly formalized system of assessment of euhydration status and propose different hydration biomarkers for assessment of hydration status.

In this study, the primary objective was to determine the water intake, hydration status, and related hydration urine and blood biomarkers among physically active male young adults. The secondary objective was to analyze the associations among water intake, hydration status, and urine and blood biomarkers. The results of the study could provide scientific and accurate references for the development of recommendations on water intake for athletes. Moreover, for athletes and the general population, the data of the study could provide more clues and references for the establishment of comprehensive evaluation indexes on hydration status [41].

## 2. Materials and Methods

### 2.1. Sample Size Calculation

The sample size (*N*) was calculated using the following formula [42]. *N*=*t*^2^(*P*(1 − *P*)/*ℓ*^2^)*t* means the corresponding statistic value when the confidence was set as 95%; that is, *α* = 0.05 and *t* = 1.96. In addition, *e* was the error value and was set as 4%. Referring to the percentage of participants with a hydrated status in all days during the study period in the previous study, *P* was set as 4.4% [35]. Considering the missed follow-up rate of 10%, 111 participants were needed.

In this study, a total of 111 participants who met the inclusion criteria were recruited. All 111 participants read and voluntarily signed their informed consent. Finally, 109 participants completed the study. Two participants dropped out due to self-withdrawal.

### 2.2. Participants

One hundred-and-eleven physically active male young adults aged 18–23 years were recruited by offline campus advertising from Beijing Sport University in Beijing, China (refer to Table 1).

The inclusion criteria were as follows: age range from 18 to 25 years, being in a healthy status and male, having more than five periods of moderate-intensity physical activity (bodybuilding, table tennis, track and field, tennis, football, and others) per week. Moderate physical activities were defined as activities with an energy expenditure of 4.0–7.0 kcal/min [1]. The number of minutes per week for this physical activity was more than 150 minutes. The exclusion criteria were as follows: having diseases of the gastrointestinal tract or of the kidney, cognitive disorders, or other chronic and metabolic disease; having a history of taking drugs, vitamins, and other health products within one month.

### 2.3. Ethics

The study protocol was reviewed and approved by the Ethical Review Committee of Peking University. The identification code is IRB 00001052-19051. The study protocol has been registered on the Chinese clinical trial registry website, and the identification code is Chi CTR 1900025710. The study was conducted in accordance with the guidelines of the Declaration of Helsinki. Prior to the beginning of the study, all the participants read and voluntarily signed their informed consent.

### 2.4. Study Design and Procedure

The cross-sectional studies study was conducted from April to May 2019. On the first day and the seventh day, the height, weight, waistline, vital capacity, and body composition of the participants were measured. From day 1 to day 7, the “7-day 24-hour fluid intake questionnaire” (liq. In 7) was used and water intake behaviors were recorded in real-time in free-living condition. Using a uniformly customized cup with a scale to the nearest 5 mL as a reference, the participants' drinking fluid intake was evaluated and recorded. From day 3 to day 5, the weighing method, duplicate portion method, and direct drying method were used to calculate the water intake from food over the 3 days, including two working days and one weekend day. In free-living conditions, participants could be free to choose their own foods. From day 3 to day 5, the “3-day 24-hour urine excretion record” was also used to record all the urine in real-time. All collected urine samples were weighed to calculate 24 h urine volume. Related urine biomarkers, including 24 h urine and first morning urine osmolality, USG, and concentration of K, Na, and Cl, were determined by researchers. On day 4, cubital venous blood was collected. Related blood biomarkers, including copeptin, blood glucose, blood lipid, testosterone, cortisol, creatinine, blood ions, and other related biomarkers, were determined. From day 1 to day 7, physical activity was recorded by participants to calculate the energy expenditure due to physical activities among the participants. In addition, data on the temperature and humidity where the participants were located for these days were measured and recorded in real time. The indicators collected in different study time points are shown in Table 2.

### 2.5. Anthropometric Measurements

The weight, height, and waistline of participants were measured by trained researchers following a standardized procedure. Wearing light clothing and no footwear, the height of participants was measured twice with 0.1 cm accuracy, and the weight of participants was measured twice with 0.1 kg accuracy, using a height–weight meter (HDM-300; Huaju, Yiwu, Zhejiang, China). Participants' waistlines were measured twice with 0.1 cm accuracy using tape. The parameters were measured in duplicate.

### 2.6. Assessment of Daily Total Drinking Fluid Intake (TDF)

The “7-day 24-hour fluid intake questionnaire” (liq. In 7) was used to evaluate the amount of fluid intake in this study, which was verified to be effective in previous studies [43, 44]. The participants were asked to record all behaviors related to fluid intake for 7 consecutive days after training (see Supplementary Table 1). The amount, type, time, and place of fluid intake were recorded in detail. To help the participants to assess the amount of fluid intake, customized cups with a scale to the accuracy of 5 mL were provided to participants as a reference. All types of water/beverages were included. The definition of water/beverage types in this study were as follows. The types of plain water included tap water, packaged or bottled mineral water and purified water. The types of milk and dairy products included pure milk, plain yogurt, and other milk products without the artificial addition of sugar in the production process. The types of sports beverages included self-made or bottled sports beverages, energy beverages, and electrolyte beverages mainly for the sports crowd. Sugar-sweetened beverages (SSBs) included beverages with the artificial addition of sugar in the process of beverage production, including carbonated beverages, flavor beverages, and other beverages that were not classified as sports beverages. The types of tea referred to self-made green tea, scented tea, black tea, and so on, excluding tea beverages with sugar added in. In addition, all behaviors related to alcohol intake have also been recorded.

The TWI recommendation for male adults set by the Chinese Nutrition Society, World Health Organization (WHO), and European Food Safety Authority (EFSA) were 3.0 L, 2.9 L, and 2.5 L, respectively [1, 45]. The TDF recommendation for male adults set by the Chinese Nutrition Society was 1.7 L [1].

### 2.7. Assessment of Daily Water Intake from Food (WFF)

All food over three consecutive days, including two working days and one weekend day, was recorded and weighted by researchers using an electronic balance (YP20001, SPC, Shanghai, China). Participants ate every meal with researchers so that food intake could be accurately weighed by researchers in a timely manner. The duplicate portion method was used in this study. All kinds of food eaten by participants were prepared in two duplicate portions, one for eating by participants and the other were for sample collection. The weight of each food before and after eating (including inedible and inedible parts, such as bones or other parts of food that were eaten) was recorded, and then, the actual amount of food intake was calculated (g). Using the method of duplicate portion sampling, the backup food samples were collected for testing [35]. The water content in food was tested using the direct drying method according to the *National Food Safety Standard GB 5009.3-2016 Determination of W*a*ter in Foods* [46]. Parallel samples were tested for each kind of food, and the error of the two test results did not exceed 5%. Then, the percentage of water in food and water intake from food were calculated [35]:

(1) $$\begin{array}{c} \text{the percentage of water in food}\left(\%\right)=\frac{\left[\text{weight of food before drying}\left(g\right)-\text{weight of food after drying}\left(g\right)\right]}{\text{weight of food before drying}\left(g\right)}\times 100\%\text{,}\\ \text{water intake from food}\left(\text{mL}\right)=\frac{\text{the amount of food intake}\left(g\right)\times \text{the percentage of water in food}\left(\%\right)}{1.0\left(g/\text{mL}\right)}\text{,}\\ \text{daily total water intake}\left(\text{TWI}\right)\left(\text{mL}\right)=\text{daily total drinking fluid intake}\left(\text{TDF}\right)\left(\text{mL}\right)+\text{daily water intake from food}\left(\text{WFF}\right)\left(\text{mL}\right)\text{.} \end{array}$$

### 2.8. Determination of Urine biomarkers

From day 3 to day 5, all participants' urine samples for each day were collected in disposable urine storage bags by participants, and then the samples were stored at +4°C no more than two hours before tests of urine biomarkers [35]. Collected urine samples, starting from the second voiding on one day and ending on the first voiding on the next day, were defined as 24 h urine. From day 3 to day 5, the first morning urine referred to the first excretion of urine after getting up in the morning, before breakfast, and before physical activity. Urine volume was measured with an electronic desktop scale (YP20001; SPC; Shanghai, China) with the accuracy 0.1 kg. Urine osmolality was determined by researchers using the osmolality weight molar concentration meter (SMC30C; Tian he; Tianjin; China) following a standardized procedure, which was accurate to 1 mOsm/kg. The principle for determining urine osmolality was the freezing point descent method. Urine-specific gravity (USG), concentration of K, Na, and Cl were determined by urine analyzer (UA-600; Mindray; Shenzhen; China), and urine test strip (U-11; Mindray; Shenzhen; China).

### 2.9. Judgment and Definition of Hydration Status

According to the 24 h urine osmolality, participants were divided into three groups as follows: groups with a euhydration status, middle hydration status and hypo hydration status. When the 24 h urine osmolality was >800 mOsm/kg, the participant was in a hypohydration status [31, 32, 47, 48]. When the 24 h urine osmolality was between 500 and 800 mOsm/kg, the participant was in a middle hydration status [31]. When the 24 h urine osmolality was <500 mOsm/kg, the participant had a euhydration status [31, 33].

### 2.10. Determination of Blood Biomarkers

Approximately 5 mL of fasting cubital venous blood of participants was collected on day 4 in this study. Serum creatinine was detected by researchers using a creatinine detection kit (C 011-2-1; Jian cheng; Nanjing; China). Serum cortisol, testosterone, and copeptin were measured using an i Mark microplate reader (i Mark; Bio-Rad; California; USA) by researchers. An automatic biochemical analyzer (Cob as C 501; Roche; Basel, Switzerland) was used by researchers to detect blood sodium, blood potassium, and blood chlorine. An automatic biochemical analyzer (Cob as C 501; Roche; Basel, Switzerland) was used to detect blood glucose and other blood indexes.

### 2.11. Calculation of Energy Expenditure of Physical Activity

Physical activity was recorded by participants using the self-made “questionnaire on sports training plan” to investigate its respective frequency and the time of major projects, gym training and other sports every week, and their average time spent sports training every day was calculated using the above indicators. “Questionnaire on sports training plan” is the training plan of participants, including daily schedule, sports type, sports duration, and some other detailed information. Metabolic equivalent (MET) is a variable that is often used to evaluate oxygen consumption and energy consumption during physical activity [49]. 1 MET is defined as the oxygen consumption of a person sitting quietly without physical activity, which is about 3.5 mL/(kg·min). When converted into energy consumption, this is about 1 kcal/(kg·h) [50]. Referring to the method in the relevant literature [8], the metabolic equivalent, corresponding to the training types of sport conducted by the participants over the week, was obtained. Referring to the method of another piece of relevant literature [9], the energy expenditure caused by physical activity among participants over one week was estimated using the training plan and weight data.

### 2.12. Temperature, Humidity, and Wind Speed

Using the temperature and humidity meter (WSB-1-H2; Exasace; Zhejiang, China), the researchers measured and recorded the temperature and humidity of the place where the participants lived at 10 a. m every day during the study period. The accuracy of temperature and humidity measurements were 1°C and 0.1% RH, respectively.

### 2.13. Quality Control

Unification of study procedure was developed before the study. Standardized training was conducted for researchers to make them familiar with all study procedure sand related methods. All participants also received unified training on the study procedure that they needed to participate in, the questionnaire content and the methods of sample collection. The quality control group was set in the study. During the entire study, all the procedures were strictly supervised by researchers in the quality control group. The completed questionnaires were double-checked in each day. The coding, storage, and testing of related samples were double-checked each day.

### 2.14. Statistical Analysis

The software of SAS 9.2 (SAS Institute Inc., Cary, NC, USA) was used for statistical analysis. For quantitative parameters in line with the normal distribution, the mean and standard deviation (SD) were used for description, while one-way variance analysis (ANOVA) was used to analyze the differences among participants with different hydration statuses. Bonferroni-corrected posthoc multiple comparisons were conducted to test for discrepancies among different hydration status. For quantitative parameters that were not in line with the normal distribution, the median and interquartile were used for description, while Kruskal–Wallis test was used to analyze the discrepancies in these indexes among participants with different hydration status. Count data were described as *n* (percentage), and a Chi-square test was used to analyze the differences in these indexes. Spearman's correlation coefficients were used to analyze the intensity of the correlations among water intake, urine, and blood biomarkers. Significance level was set to be less than *P* of 0.05.

## 3. Results

### 3.1. Participants Characteristics and the Environment

In this study, a total of 111 participants who met the inclusion criteria were recruited. Finally, 109 participants completed the study, and 2 participants dropped out due to self-withdrawal. The 109 participants' characteristics are shown in Table 1.

According to the 3-day, 24-hour urine osmolality, 16% of participants had a euhydration status, 43% of participants had a hypohydration status, and 41% of participants had a middle hydration status. There was no statistical significance regarding the characteristics of participants in different hydration group, except for age (*χ*^2^ = −7.141, *P*=0.01) (Table 1).

The average temperature was 24.2°C ± 5.8°C at 10 a. m. every day, and the average humidity was 29.5% ± 15.8% RH during the study.

### 3.2. Water Intake

Among the 109 participants, the median daily TWI, TDF, and WFF were 2701 mL, 1789 mL, and 955 mL, respectively. The percentages of participants whose TWI and TDF met the TWI recommendation for male adults set by the Chinese Nutrition Society were 38.5% and 55.0%, respectively. According to the percentage of different sources in TDF, the top three sources were plain water, SSBs, and sports beverages, accounting for 67.1%, 22.2%, and 3.7% of the total. The top three sources of WFF were dishes, staple food, and soup and porridge, accounting for 46.1%, 37.2%, and 10.0%.

There were statistical significances in TWI, TDF, and plain water among participants with a different hydration status (*χ*^2^ = 16.526, *P* < 0.001; *χ*^2^ = 15.395, *P* < 0.001; *χ*^2^ = 26.021, *P* < 0.001). Statistical significances were also found in the percentages of plain water, sports beverages, and dairy products in TDF of participants with a different hydration status (*χ*^2^ = 15.324, *P* < 0.001; *χ*^2^ = 6.276, *P* < 0.043; *χ*^2^ = 6.054, *P*=0.048). The percentages of participants whose TDF met the recommendation on TWI for male adults set by the Chinese Nutrition Society showed statistical differences in different hydration statuses (*χ*^2^ = 6.988, *P*=0.030; *χ*^2^ = 10.533, *P*=0.005; *χ*^2^ = 8.223, *P*=0.016) (Table 3).

### 3.3. Urine Biomarkers

There were statistical significances in the 24 h urine volume, osmolality, USG, and concentrations of K, Na, and Cl among participants with different hydration statuses. Statistical significances were also found in terms of the morning urine osmolality, USG, and concentrations of K, Na, and Cl among participants with different hydration statuses (Table 4).

### 3.4. Blood Biomarkers

Among 109 participants, the median copeptin was 1.6 pmol/L. The blood glucose, TC, TG, LDL-C, and HDL-C were 4.1, 5.1, 0.8, 2.4, and 1.6 mmol/L, respectively. The concentration of testosterone, cortisol, and creatinine were 16.7 nmol/L, 82.7 ng/mL, and 65.0 *μ*moI/L, respectively. The concentration of blood K, Na, and Cl ion were 4.2, 139.8, and 102.9 mmol/L, respectively. The Leukocyte count, red blood cell count, hemoglobin concentration, hematocrit, mean red blood cell volume, mean hemoglobin content, platelet count, mean platelet volume, platelet hematocrit, neutrophil absolute value, and percentage of neutrophils were 6.3 × 10^9^/L, 5.1 × 10^12^/L, 152.0 g/L, 55.2%, 108.3 fL, 29.4 pg, 253.0 × 10^9^/L, 11.1 fL, 0.3%, 3.0 × 10^9^/L, and 47.3%, respectively. No statistical significance was found in the above blood biomarkers (Table 5).

### 3.5. Correlations between Water Intake, Urine and Blood Biomarkers

Moderate-intensity positive correlations were found between the TWI and 24 h urine volume (*r* = 0.408, *P* < 0.001). Moderate-intensity negative correlations were found between the TWI and 24 h urine osmolality, 24 h urine Na concentration, morning urine osmolality, and morning urine Na concentration (*P* < 0.01 for all).

There were also moderate-intensity negative correlations between TDF and 24 h urine osmolality, morning urine osmolality, and morning urine Na concentration (*P* < 0.01 for all). Increased and higher moderate-intensity positive correlations were found between plain water and 24 h urine volume (*r* = 0.374, *P* < 0.001). Increased and higher moderate-intensity negative correlations were found between plain water and 24 h urine osmolality, 24 h urine K concentration, 24 h urine Na concentration, morning urine osmolality, and morning urine Na concentration (*P* < 0.01 for all). The correlation between sports beverages and 24 h urine osmolality, USG and 24 h urine Na concentration was low but positive (*r* = 0.218, *P*=0.023; *r* = 0.206, *P*=0.031; *r* = 0.194, *P*=0.043), in contrast to that of TWI, TDF and plain water (Table 6).

There were moderate-intensity positive correlations between WFF and 24 h urine volume (*r* = 0.366, *P* < 0.001). Moderate-intensity positive correlations were found between water intake from staple food and 24 h urine volume (*r* = 0.327, *P*=0.001).

## 4. Discussion

In this study, the median TDF of the participants was 1789 ml, the median TWI was 2701 mL, and the proportion of TDF to TWI was 65.0%, which was higher than the corresponding results in the previous study, conducted with young adults in China (1214 mL, 2483 mL, and 48.9%) [35, 51]. In this study, 56.0% of the participants reached the recommendations for adequate TDF for Chinese male adults (1700 mL), and 37.6% reached the recommendations for adequate TWI for Chinese male adults (3000 mL) [1], which was also higher than the corresponding results in the previous study conducted with young adults in China (23.5% and 25.0%) [35, 51]. The percentages of participants whose TWI met the TWI recommendation for male adults set by the World Health Organization (WHO) and European Food Safety Authority (EFSA) were 42.2% and 68.8%, respectively. In some studies abroad, the TWI of men undergoing physical activity training ranged from 3.2 to 10.3 L per day, which was also higher than the TWI of nonathlete [52]. Athletes with a high level of physical activity also have a high demand for water intake and should drink an adequate amount of water.

This study found that plain water was the main source of TDF for participants, which was similar to the results gathered among adults and children in China [32–34]. Sports beverages and SSBs were another two important sources of TDF for participants in this study. A 24-hour retrospective study in Russia found that athletes mainly drink bottled water, followed by tea, and only some athletes drank sports beverages during cyclical sports and single combat [35]. Compared with athletes competing for indifferent types of sports, 95∼96% of athletes with single-confrontation and other strength sports drank bottled water, while the proportion of athletes in other sports who drank bottled water was lower, at about 67%–79% [35]. The TDF of the martial arts group was the highest, at about 2326 mL, while that of the complex coordinated physical activity group was the lowest, at about 1009 mL [35]. Among the participants, 76% of athletes ate liquid food outside the training period, and the WFF was between 382 mL and 553 mL [35].

In this study, the median 24-hour urine volume of the participants was 850 mL, which was lower than the corresponding result in a previous study conducted among young adults in China (1272 mL) [35, 51]. This may be because participants in this study were athletes with high-intensity physical activity and more water was lost through sweat. In this study, judging by 24 h urine osmolality, only 16% of the participants were in the hydration status and 43% were in hypohydration status. Compared with the results of the previous study conducted among young adults in China, the proportion of athletes with hydration status was lower and the proportion with hypohydration status was higher [51]. There are no further studies on the water intake and hydration status of athletes in daily life in China. Hypohydration caused by insufficient water intake is common in athletes. In 2009, one study collected the urine samples of 138 athletes from the University of New England and analyzed the hydration status by measuring their urine-specific gravity (USG). The results of this study showed that 13% of participants were in a hypo-hydration status, and the proportion of participants in a hypo-hydration status (47%) was higher than that of female athletes (28%) [25]. In 2015, one survey conducted among 96 male basketball players from eight national teams found that more than 75% of the players were hypohydrated before the game and continued to be hypohydrated during the game [26]. In 2012, one study was conducted among 21 young male professional football players in a cold environment, and the results showed that 14 athletes were in a hypohydration status before training [27]. In 1999, 98 participants were recruited for long-distance walking in one study. During walking, the participants could drink water at any time, after that, their weight loss was measured. The results showed that the participants had varying degrees of water loss. Among them, the average water loss of men (1.6% body weight) was significantly higher than that of women (0.9% body weight). The percentage of participants that were hypohydration (water loss of more than 2% of body weight) was also higher in men (34%) than in women (12%), indicating that even if water can be replenished during physical activity, athletes may still be hypohydration during physical activity, and male athletes were more likely to be hypohydration [53]. In 2009, one study examined the urine samples of 29 male players in a professional basketball league before the game and monitored their water loss during the game. The results showed that about half of the players were hypohydrated before the game, and the water supplementation in the game did not change the hypohydration status before the game [54]. In 2015, another survey with male young basketball players participating in the U20 European Champions League found that more than 75% of the players were hypohydrated before the game, and hydration status was determined using both urine specific gravity and percent loss of body weight [26, 30]. The relevant studies abroad mainly focused on hydration status before, during, and after physical activity; data on water intake and hydration in daily life are lacking. In addition, all the studies discussed in this section that relied on urine osmolality or urine specific gravity are potentially overestimating the percentage of athletes who are hypohydrated.

The decrease in TWI, TDF, and 24 h urine volume is correlated to the increase in the degree of hypo hydration. There was no significant difference in blood indexes. The study found that the hydration status of the participants may be closely related to their water intake, urination behavior, and urine indicators. Similar results were found in the research on young people in China [35, 51]. The result of one study abroad found strong associations (|*r*| ≧ 0.6) between TDF and 24 h urine volume, osmolality, and USF among adults [3]. Another study conducted among pregnant and lactating women reported a significant relationship between total fluid intake and urine volume (*r* = 0.71) [55]. Some studies showed that some blood indexes were also good markers for the assessment of hypohydration status, but were insensitive to mild hydration status in daily life [40], which was similar to the results of this study. Copeptin, the c-terminal portion of arginine vasopressin, which may be related to metabolic syndrome through altering insulin and glucagon secretion, was determined in this study. The blood glucose and insulin levels of the participants were normal, without abnormality, according to the inclusion and exclusion criteria. No difference was found among participants with different hydration statuses.

This study also found some interesting results that need to be considered. Increased and higher moderate-intensity correlations were found between plain water and 24 h urine volume, 24 h urine osmolality, 24 h urine K concentration, 24 h urine Na concentration, morning urine osmolality, and morning urine Na concentration. The intensity of correlations between plain water and urine indexes were higher than those of TWI, TDF, and WFF. The correlations between sports beverages and 24 h urine osmolality, USG, and 24 h urine Na concentration were low but positive, in contrast to those of TWI, TDF, and plain water. The results show that different types of water/beverages had different effects on the hydration status and plain water had a better effect on improving hydration status. One study found a similar result: supplementation with soft drink-like beverages exacerbated hypo hydration [56]. One study examined the potential effects of different beverages on hydration status, and the results found that the total urine mass over 4 hours after oral rehydration solution, full-fat milk, and skimmed milk were smaller than that of still water [57, 58], but cola was no worse than water or sports drinks when it came fluid retention after 4 hours [58]. However, one study showed different results that those with sugar (juices and sodas), actually have pretty good hydration properties [58]. The results of the above studies showed that, when establishing a water-replenishment strategy to improve hydration status, we should not only pay attention to the water intake, but also consider the impacts of different water/beverage types on hydration status.

This study has some strengths and weaknesses. This was the first study to analyze the water intake and hydration status of physically active male young adults in daily life in China. In consideration of the study's weaknesses, a larger sample would provide more representative data. Only male adults were studied, and data on physically active female young adults are lacking. In addition to only men participating, only healthy, active, and young men participated which limits the populations to which the results can be applied. Only urine osmolality thresholds were used to define hypo hydration in this study, which is dependent on body size and muscle mass. People with larger amounts of muscle mass excrete more creatinine and other muscle metabolites in their urine. As result, larger individuals are more likely to be misclassified as dehydrated when they may not actually be. It would be better to use more comprehensive indexes for the assessment of hydration status. In addition, body composition should be measured more times, which could be useful in determining the effects of lean mass on urine osmolality. Further, no measurements of mood and health were taken in this study. Additionally, this study was an observational study. In the future, it will be very useful to conduct related studies with larger samples and to investigate the amount of extracellular and intercellular body water.

## 5. Conclusions

In summary, the percentages of participants whose TWI and TDF met the related recommendation for male adults set by the Chinese Nutrition Society were 38.5% and 55.0%. Moreover, 43% of participants were in hypo hydration status when evaluated using urine osmolality. The more comprehensive index should be used to further analyze functional hypohydration. The amount and types of water intake were correlated with hydration status and urine biomarkers. There are few studies on water intake among the physically active population and athletes. More detailed and targeted surveys are needed to obtain basic data on water intake. It is necessary to carry out long-term and routine monitoring of hydration status among the physically active population and athletes. These data are vital for the development of recommendations on adequate water intake among the physically active population and athletes and the establishment of water-replenishment schemes and measures during physical activity and daily life.

## Figures and Tables

**Table 1:** Characteristics of participants.

	Total	Euhydration status	Middle hydration	Hypo hydration	*χ* ^2^	*P*
	*n* = 109	*n* = 17	*n* = 45	*n* = 47		
Age (y)	21.0 (1.0)	21.0 (1.0)	21.0 (1.0)	20.0 (1.0)	7.141	0.028^*∗*^
Height (cm)	178.7 (6.8)	182.1 (9.1)	178.7 (5.8)	178.3 (6.1)	3.056	0.217
Weight (kg)	69.7 (9.5)	69.2 (9.7)	69.7 (10.2)	69.7 (9.1)	0.592	0.744
BMI (kg/m^2^)	22.0 (2.4)	21.1 (2.9)	22.2 (2.7)	22.3 (2.2)	3.723	0.155
Skeletal muscle (kg)	34.7 (4.2)	34.3 (4.3)	34.7 (5.3)	35.1 (3.5)	1.294	0.524
Body fat (k)	9.1 (4.9)	10.3 (5.7)	8.9 (6.9)	9.3 (3.8)	0.241	0.886
Percentage of body fat (%)	12.8 (5.9)	15.2 (6.1)	12.7 (7.9)	12.6 (4.2)	0.583	0.747
Waistline (cm)	75.4 (6.5)	75.4 (5.8)	74.9 (6.6)	75.5 (6.7)	0.267	0.875
Physical activity (kcal/w)	5843 (5699)	6539 (6489)	5724 (5716)	5869 (6119)	0.229	0.892

Values are shown as the median (interquartile). Kruskal–Wallis test was used to analyze the discrepancy of these indexes among participants in different hydration status. ^*∗*^, statistically significant differences, *P* < 0.05.

**Table 2:** The indicators were collected at different time points in the study.

	Day 1	Day 2	Day 3	Day 4	Day 5	Day 6	Day 7
Individual information	√						
Physical measurement	√						√
7-day 24-hour fluid-intake questionnaire	√	√	√	√	√	√	√
Water intake from food			√	√	√		
Blood biomarkers				√			
24 h urine and related biomarkers			√	√	√		
Physical activity	√	√	√	√	√	√	√
Environment	√	√	√	√	√	√	√

√, the measure was taken on that day.

**Table 3:** Water intake of participants with different hydration statuses.

	Total	Euhydrationstatus	Middle hydration	Hypohydration	*χ* ^2^	*P*
	*n* = 109	*n* = 17	*n* = 45	*n* = 47		
Daily TWI (mL)^a^	2701 (974)	3414 (1466)	2701 (778)	2532 (1115)	16.526	<0.001^*∗*^
Percent meet China water AI (%)^b^	42 (38.5)	11 (64.7)	16 (35.6)	15 (31.9)	5.954	0.051
Percent meet WHO water AI (%)^b^	46 (42.2)	12 (70.6)	18 (40.0)	16 (34.0)	6.988	0.030^*∗*^
Percent meet the EFSA AI (%)^b^	75 (68.8)	17 (100.0)	31 (68.9)	27 (57.4)	10.533	0.005

Daily TDF (mL)^a^	1789 (863)	2586 (1229)	1800 (615)	1579 (926)	15.395	<0.001^*∗*^
Percent meet China water AI (%)^b^	60 (55.0)	14 (82.4)	26 (57.8)	20 (42.6)	8.223	0.016^*∗*^
Percent of TWI (%)^a^	65.0 (15.0)	71.7 (12.3)	65.1 (15.9)	62.7 (14.1)	4.796	0.091
Sources of TDF						
Plain water						
Amount (mL)^a^	1181 (666)	1538 (868)	1286 (607)	888 (593)	26.021	<0.001^*∗*^
Percent (%)^a^	67.1 (22.8)	74.9 (21.0)	69.4 (24.5)	58.6 (22.8)	15.324	<0.001^*∗*^
SSBs						
Amount (mL)^a^	383 (365)	429 (489)	397 (361)	351 (353)	0.011	0.994
Percent (%)^a^	22.2 (16.9)	19.4 (15.9)	22.2 (17.5)	23.2 (19.0)	4.702	0.095
Sports beverages						
Amount (mL)^a^	65 (154)	62 (91)	36 (129)	86 (214)	3.628	0.163
Percent (%)^a^	3.7 (7.4)	3.2 (4.5)	1.3 (6.4)	5.7 (10.5)	6.276	0.043^*∗*^
Dairy products						
Amount (mL)^a^	40 (111)	31 (71)	34 (106)	57 (169)	3.444	0.179
Percent (%)^a^	2.2 (6.7)	1.0 (3.6)	1.8 (5.8)	3.2 (9.4)	6.054	0.048^*∗*^
Tea						
Amount (mL)^a^	0 (0)	0 (0)	0 (0)	0 (0)	0.413	0.813
Percent (%)^a^	0.0 (0.0)	0.0 (0.0)	0.0 (0.0)	0.0 (0.0)	0.395	0.821

Daily WFF (mL)^a^	955 (472)	1055 (303)	953 (488)	939 (467)	1.823	0.402
Percent of TWI (%)^a^	35.0 (15.0)	28.3 (12.3)	34.9 (15.9)	37.3 (14.1)	4.796	0.091
Sources of WFF						
Dishes						
Amount (mL)^a^	454 (213)	507 (207)	458 (190)	427 (273)	5.015	0.081
Percent (%)^a^	46.1 (14.8)	50.8 (13.5)	49.1 (14.0)	43.9 (16.9)	4.769	0.092
Staple food						
Amount (mL)^a^	330 (106)	387 (147)	316 (123)	330 (84)	2.230	0.328
Percent (%)^a^	37.2 (12.4)	38.8 (10.4)	36.3 (11.6)	37.7 (16.2)	0.035	0.982
Soup and porridge						
Amount (mL)^a^	89 (177)	89 (114)	65 (195)	112 (198)	0.456	0.796
Percent (%)^a^	10.0 (16.0)	9.4 (11.2)	7.4 (16.2)	10.5 (18.1)	0.809	0.667
Snacks						
Amount (mL)^a^	9 (64)	10 (54)	2 (63)	13 (69)	0.556	0.757
Percent (%)^a^	1.1 (5.9)	1.1 (4.7)	0.3 (5.8)	1.2 (6.7)	0.681	0.711

^a^Values are shown as the median (interquartile). Values were compared using Kruskal–Wallis test, ^b^values were shown as *n* (percentage), and values were compared using Chi-square test; ^*∗*^statistically significant differences, *P* < 0.05. TWI: daily total water intake. TDF: daily total drinking fluid intake. WFF: daily water intake from food. SSBs: sugar-sweetened beverages. WHO means World Health Organization (WHO). EFSA: European food safety authority (EFSA). The TWI recommendation for male adults set by the Chinese nutrition society, World Health Organization (WHO), and the European Food Safety Authority (EFSA) were 3.0 L, 2.9 L, and 2.5 L, respectively. The TDF recommendation for male adults set by the Chinese nutrition society was 1.7 L. AI means recommendations on adequate intake.

**Table 4:** Urine biomarkers of participants with different hydration statuses.

	Total	Euhydration status	Middle hydration	Hypohydration	*χ* ^*2*^	*P*
	*n* = 109	*n* = 17	*n* = 45	*n* = 47		
24 h urine volume (mL)	850.2 (408.0)	1093.0 (709.1)	896.5 (456.6)	640.5 (292.2)	28.212	<0.001^*∗*^
24 h urine						
Osmolality (mOsm/kg)	764.0 (286.7)	436.3 (111.1)	671.3 (148.0)	903.0 (114.3)	91.341	<0.001^*∗*^
USG	1.020 (0.007)	`1.015 (0.005)	1.018 (0.007)	1.023 (0.005)	47.721	<0.001^*∗*^
K (mmol/L)	45.2 (12.9)	33.4 (9.4)	41.3 (11.8)	49.2 (7.7)	41.548	<0.001^*∗*^
Na (mmol/L)	201.6 (66.4)	147.2 (32.5)	188.0 (50.5)	234.3 (57.8)	46.863	<0.001^*∗*^
Cl (mmol/L)	221.1 (53.2)	170.8 (20.8)	217.8 (42.3)	235.7 (32.6)	40.839	<0.001^*∗*^

First morning urine						
Osmolality (mOsm/kg)	862.3 (271.3)	499.0 (328.7)	754.3 (226.2)	922.3 (196.0)	52.834	<0.001^*∗*^
USG	1.027 (0.005)	1.022 (0.004)	1.027 (0.007)	1.028 (0.005)	18.567	<0.001^*∗*^
K (mmol/L)	50.6 (12.6)	37.7 (17.1)	47.0 (16.0)	52.0 (12.5)	11.439	<0.001^*∗*^
Na (mmol/L)	197.1 (57.7)	144.0 (43.5)	182.9 (43.8)	222.8 (41.1)	37.687	<0.001^*∗*^
Cl (mmol/L)	203.5 (42.4)	164.2 (42.1)	202.2 (37.9)	212.4 (40.1)	23.786	<0.001^*∗*^

Values were shown as the median (interquartile). Values were compared using the Kruskal–Wallis test. ^*∗*^statistically significant differences, *P* < 0.05. USG means urine-specific gravity.

**Table 5:** Blood biomarkers of participants in different hydration statuses.

	Total	Optimal hydration	Middle hydration	Dehydration	*χ* ^*2*^	*P*
	*n* = 109	*n* = 17	*n* = 45	*n* = 47		
Copeptin (pmol/L)	1.6 (0.2)	1.6 (0.1)	1.6 (0.2)	1.7 (0.2)	0.990	0.610
Blood glucose (mmol/l)	4.1 (0.9)	4.6 (1.4)	4.0 (1.2)	4.1 (1.0)	5.390	0.068
Blood lipid						
TC (mmol/L)	5.1 (1.2)	5.1 (1.3)	5.0 (0.8)	5.1 (1.7)	2.669	0.263
TG (mmol/L)	0.8 (0.4)	0.8 (0.4)	0.9 (0.4)	0.8 (0.5)	1.565	0.457
LDL-c (mmol/L)	2.4 (0.6)	2.6 (0.7)	2.4 (0.6)	2.4 (0.7)	0.051	0.975
HDL-c (mmol/L)	1.6 (0.5)	1.7 (0.4)	1.6 (0.4)	1.7 (0.5)	1.768	0.413
Testosterone (nmol/L)	16.7 (2.8)	15.1 (3.0)	16.6 (2.6)	17.1 (2.5)	1.542	0.463
Cortisol (ng/mL)	82.7 (21.5)	77.3 (34.7)	81.5 (24.5)	83.1 (19.8)	0.955	0.620
Creatinine (*μ*moI/L)	65.0 (19.5)	66.7 (21.7)	66.7 (28.2)	65.0 (21.7)	2.396	0.302
Blood ions						
K (mmol/L)	4.2 (0.9)	4.5 (1.3)	4.1 (0.8)	4.3 (0.9)	3.747	0.154
Na (mmol/L)	139.8 (6.3)	139.0 (6.3)	139.2 (6.4)	140.2 (6.2)	2.050	0.359
Cl (mmol/L)	102.9 (10.2)	102.1 (9.2)	102.9 (9.3)	102.9 (12.8)	0.287	0.867
Leukocyte count (10^9^/L)	6.3 (2.0)	6.4 (1.8)	6.7 (2.4)	6.2 (2.0)	3.568	0.168
Red blood cell count (10^12^/L)	5.1 (0.5)	5.2 (0.5)	5.1 (0.6)	5.1 (0.5)	0.018	0.991
Hemoglobin concentration (g/L)	152.0 (11.5)	153.0 (11.0)	152.0 (12.5)	151.0 (12.0)	0.694	0.707
Hematocrit (%)	55.2 (4.5)	56.3 (4.5)	54.5 (4.2)	54.7 (4.9)	0.408	0.815
Mean red blood cell volume (fL)	108.3 (4.0)	108.4 (2.5)	108.5 (4.8)	107.9 (4.1)	0.170	0.919
Mean hemoglobin content (pg)	29.4 (2.0)	29.6 (2.3)	29.3 (2.5)	29.4 (1.9)	0.201	0.904
Platelet count (10^9^/L)	253.0 (55.0)	258.0 (86.0)	258.0 (52.5)	246.0 (58.0)	2.303	0.316
Mean platelet volume (fL)	11.1 (1.0)	11.4 (1.0)	11.0 (0.9)	11.1 (1.0)	3.062	0.216
Platelet hematocrit (%)	0.3 (0.1)	0.3 (0.1)	0.3 (0.1)	0.3 (0.1)	2.617	0.270
Neutrophil absolute value (10^9^/L)	3.0 (1.4)	2.7 (1.1)	3.2 (1.7)	3.0 (1.0)	2.904	0.234
Percentage of neutrophils (%)	47.3 (12.0)	42.2 (16.5)	49.5 (14.6)	47.2 (6.7)	2.042	0.360

Values were shown as the median and interquartile; ^*∗*^, statistically significant differences, *P* < 0.05. TC means total cholesterol. TG means triglyceride. LDL-C means low-density lipoprotein cholesterol. HDL-C means high-density lipoprotein cholesterol.

**Table 6:** Correlations between water intake and urine biomarkers.

			TDF (mL)^a^								WFF							
	TWI (mL)		Total		Plain water		SSBs		Sports beverages		Total		Dishes		Staple food		Soup and porridge	
	*r*	*P*	*r*	*P*	*r*	*P*	*r*	*P*	*r*	*P*	*r*	*P*	*r*	*P*	*r*	*P*	*r*	*P*
24 h urine volume (mL)	0.408	<0.001^*∗*^	0.278	0.003^*∗*^	0.374	<0.001^*∗*^	−0.115	0.235	0.006	0.952	0.366	<0.001^*∗*^	0.292	0.002^*∗*^	0.327	0.001^*∗*^	0.173	0.071
24 h urine																		
Osmolality (mOsm/kg)	−0.378	<0.001^*∗*^	−0.365	<0.001^*∗*^	−0.520	<0.001^*∗*^	0.046	0.633	0.218	0.023^*∗*^	−0.127	0.188	−0.166	0.084	−0.131	0.174	0.018	0.856
USG	−0.190	0.048^*∗*^	−0.119	0.219	−0.257	0.007^*∗*^	0.070	0.468	0.206	0.031^*∗*^	-0.166	0.084	−0.197	0.040^*∗*^	−0.061	0.527	0.063	0.518
K (mmol/L)	−0.193	0.044^*∗*^	−0.203	0.034^*∗*^	−0.312	0.001^*∗*^	0.111	0.249	0.118	0.222	−0.032	0.741	−0.068	0.485	0.099	0.305	0.064	0.510
Na (mmol/L)	−0.325	0.001^*∗*^	−0.284	0.003^*∗*^	−0.355	<0.001^*∗*^	−0.054	0.580	0.194	0.043^*∗*^	−0.159	0.099	−0.227	0.017^*∗*^	−0.223	0.020^*∗*^	−0.035	0.716
Cl (mmol/L)	−0.279	0.003^*∗*^	−0.245	0.010^*∗*^	−0.281	0.003^*∗*^	−0.027	0.779	0.100	0.300	−0.132	0.171	−0.200	0.037^*∗*^	−0.152	0.114	0.060	0.534
First morning urine																		
Osmolality (mOsm/kg)	−0.344	<0.001^*∗*^	−0.371	<0.001^*∗*^	−0.446	<0.001^*∗*^	0.084	0.384	0.076	0.435	−0.086	0.376	−0.129	0.180	0.029	0.763	−0.003	0.973
USG	−0.101	0.298	−0.129	0.182	−0.195	0.042^*∗*^	0.059	0.541	0.041	0.669	0.018	0.855	−0.024	0.807	0.163	0.091	0.043	0.657
K (mmol/L)	−0.114	0.239	−0.131	0.174	−0.227	0.018^*∗*^	0.125	0.194	0.084	0.386	−0.005	0.957	0.000	0.997	0.210	0.028^*∗*^	−0.047	0.625
Na (mmol/L)	−0.329	<0.001^*∗*^	−0.322	0.001^*∗*^	−0.378	<0.001^*∗*^	0.024	0.805	0.125	0.194	−0.151	0.116	−0.167	0.082	−0.135	0.162	−0.075	0.440
Cl (mmol/L)	−0.174	0.070	−0.147	0.127	−0.214	0.026^*∗*^	0.011	0.906	0.142	0.141	−0.130	0.177	−0.140	0.146	−0.094	0.329	−0.100	0.301

Spearman's correlation coefficients were used to analyze the intensity of the correlations. ^*∗*^, statistically significant differences, *P* < 0.05. TWI: daily total water intake. TDF: daily total drinking fluid intake. WFF: daily water intake from food. SSBs: sugar-sweetened beverages. USG means urine-specific gravity.

## Data Availability

The corresponding author will provide the data in a de-identified form used in the manuscript, code book, and analytic code, which is available to editors upon request either before or after publication.

## Supplementary Materials

Supplementary Materials “7-day 24-hour fluid intake questionnaire” is included. (liq. In 7).
