# Supplementary material for: Behaviors of Water Intake, Hydration Status, and Related Hydration Biomarkers among Physically Active Male Young Adults in Beijing, China: A Cross-Sectional Study
Source: Int J Clin Pract. 2022 Oct 17;2022:9436186. doi: 10.1155/2022/9436186 (PMC9592216; doi:10.1155/2022/9436186)
Supplement: Supplementary Materials — “7-day 24-hour fluid intake questionnaire” is included. (liq. In 7). [file 9436186.f1.doc]

**Supplementary table 1. “7-day 24-hour fluid intake questionnaire“ (liq. In7)**

**The first day**

|  | | **Time** | **Type** | **Place** | **Amonubt(g/mL)** |
| --- | --- | --- | --- | --- | --- |
| **Morning** | **Before breakfast** |  |  |  |  |
|  |  |  |  |  |  |
|  |  |  |  |  |  |
|  | **Breakfast** |  |  |  |  |
|  |  |  |  |  |  |
|  |  |  |  |  |  |
|  | **After Breakfast** |  |  |  |  |
|  |  |  |  |  |  |
|  |  |  |  |  |  |
| **Afternoon** | **Lunch** |  |  |  |  |
|  |  |  |  |  |  |
|  |  |  |  |  |  |
|  | **After**  **lunch** |  |  |  |  |
|  |  |  |  |  |  |
|  |  |  |  |  |  |
| **Evening** | **Dinner** |  |  |  |  |
|  |  |  |  |  |  |
|  |  |  |  |  |  |
|  | **After**  **Dinner** |  |  |  |  |
|  |  |  |  |  |  |
|  |  |  |  |  |  |
| **During sleep (before getting up)** | |  |  |  |  |
|  |  |  |  |  |  |
|  |  |  |  |  |  |
